# Comparison of Knee Pain and Difficulty With Kneeling Between Patellar Tendon and Hamstring Tendon Autografts After Anterior Cruciate Ligament Reconstruction: A Study From the New Zealand ACL Registry

**DOI:** 10.1177/03635465231198063

**Published:** 2023-09-29

**Authors:** Richard Rahardja, Hamish Love, Mark G. Clatworthy, Simon W. Young

**Affiliations:** † Department of Surgery, Faculty of Medical and Health Sciences, University of Auckland, Auckland, New Zealand; ‡ Forte Sports, Christchurch, New Zealand; § Department of Orthopaedic Surgery, Middlemore Hospital, Auckland, New Zealand; ‖ Department of Orthopaedic Surgery, North Shore Hospital, Auckland, New Zealand; Investigation performed at New Zealand ACL Registry, Christchurch, New Zealand

**Keywords:** anterior cruciate ligament, ACL reconstruction, patellar tendon graft, BTB graft, hamstring tendon graft, donor-site morbidity

## Abstract

**Background:**

The bone–patellar tendon–bone (BTB) autograft is associated with difficulty with kneeling after anterior cruciate ligament (ACL) reconstruction; however, it is unclear whether it results in a more painful or symptomatic knee compared with the hamstring tendon autograft.

**Purpose:**

To identify the rate and risk factors for knee pain and difficulty with kneeling after ACL reconstruction.

**Study Design:**

Cohort study; Level of evidence, 3.

**Methods:**

Primary ACL reconstruction procedures prospectively recorded in the New Zealand ACL Registry from April 2014 to May 2021 were analyzed. The Knee injury and Osteoarthritis Outcome Score (KOOS) was used to identify patients reporting consequential knee pain (CKP), defined as a KOOS Pain subscore of ≤72 points, and severe kneeling difficulty (SKD), defined as a self-report of “severe” or “extreme” difficulty with kneeling. Absolute values of the KOOS Pain and Symptoms subscales were also compared.

**Results:**

A total of 10,999 patients were analyzed. At 2-year follow-up, 9.3% (420/4492) reported CKP, and 12.0% (537/4471) reported SKD. The most important predictor of CKP at 2-year follow-up was having significant pain before surgery (adjusted odds ratio, 4.10; *P* < .001). The most important predictor of SKD at 2-year follow-up was the use of a BTB autograft rather than a hamstring tendon autograft (21.3% vs 9.4%, respectively; adjusted odds ratio, 3.12; *P* < .001). There was no difference between the BTB and hamstring tendon grafts in terms of CKP (9.9% vs 9.2%, respectively; *P* = .494) or in absolute values of the KOOS Pain (mean, 88.7 vs 89.0, respectively; *P* = .37) and KOOS Symptoms (mean, 82.5 vs 82.1, respectively; *P* = .49) subscales.

**Conclusion:**

At 2-year follow-up after primary ACL reconstruction, 9.3% of patients reported CKP, and 12.0% reported SKD. The BTB autograft was associated with difficulty with kneeling, but it did not result in a more painful or symptomatic knee compared with the hamstring tendon autograft.

Reconstruction of the anterior cruciate ligament (ACL) is an effective surgical procedure that restores stability to the ACL-deficient knee and allows patients to return to full activities, including pivoting sports. However, it is a major procedure that involves harvesting a graft and drilling tunnels. Avoiding complications such as graft ruptures and residual laxity is critical to the success of surgery in elite athletes. However, general complications such as persistent knee pain and an inability to kneel may be common and affect all patients undergoing ACL reconstruction.

The bone–patellar tendon–bone (BTB) and hamstring tendon autografts are the most commonly used grafts in ACL reconstruction.^1,7,8,31^ The BTB autograft is associated with a lower failure rate and a higher rate of return to activity but may be associated with anterior knee pain and difficulty with kneeling after surgery compared with the hamstring tendon autograft.^¶^ As a result, the choice of a graft is often individualized to the needs of the patient, and the benefits of a safer return to sports must be balanced against the potential consequence of knee pain.

The Knee injury and Osteoarthritis Outcome Score (KOOS) is a patient-reported outcome measure that evaluates how symptomatic or painful the knee is after ACL reconstruction.^24^ As part of the questionnaire, patients are asked whether they have any difficulty with kneeling on the reconstructed knee. Although it is suggested that the BTB autograft is associated with anterior knee pain,^1,3,6,12,26,28,35^ there are few studies with a large patient population that have directly compared the rate of knee pain between the BTB and hamstring tendon autografts. Furthermore, studies have failed to differentiate consequential knee pain (CKP) from difficulty with kneeling.^15^ Therefore, this study aimed to use prospective data recorded in the New Zealand ACL Registry to identify the proportion of patients who have CKP versus severe kneeling difficulty (SKD) after ACL reconstruction and whether the type of graft influences the rate of these outcomes.

## Methods

### New Zealand ACL Registry

The New Zealand ACL Registry is a nationwide registry that began in 2014 and prospectively captures data on patient, surgical, and follow-up variables. Since 2017, it is mandatory for all orthopaedic surgeons who perform ACL reconstruction to actively participate in the registry to achieve recertification.^17^ As of 2018, based on comparisons with government health care data, it was estimated that approximately 85% of all primary ACL reconstruction procedures performed in New Zealand were captured by the registry.^18^ Patient demographic data are collected through a preoperative self-reported questionnaire. An operative data form detailing each reconstruction procedure is completed by the surgeon. KOOS scores are collected by the registry preoperatively and postoperatively at 6 months, 1 year, and 2 years.

### Ethics

This study received exemption from Health and Disability Ethics Committee review as an audit activity. The operation of the registry has been declared a protected quality assurance activity by the New Zealand Ministry of Health, and all patients recorded in the registry have signed consent forms to participate.

### Patient Population and Inclusion Criteria

Any procedure recorded in the New Zealand ACL Registry from April 2014 to May 2021 was eligible for analysis, allowing for a minimum follow-up of 6 months. Patients who underwent multiligament reconstruction, osteotomy, unicompartmental knee replacement, or revision or contralateral ACL reconstruction before follow-up were excluded.

### Outcomes of Interest

There were 2 primary outcomes analyzed:

1. CKP: defined as a score of ≤72 points on the KOOS Pain subscale
2. SKD: defined as a self-report of “severe” or “extreme” difficulty when kneeling

CKP was defined using a cutoff score of ≤72 points on the KOOS Pain subscale, as this was a definition proposed by the Multicenter Orthopaedic Outcomes Network (MOON) group to describe significant knee pain after ACL reconstruction.^34^ As part of the Sport and Recreation subscale of the KOOS, patients were asked to rate the difficulty that they had with kneeling as either “none,”“mild,”“moderate,”“severe,” or “extreme.” SKD was therefore defined according to how it was reported. Both primary outcomes were analyzed at 6-month, 1-year, and 2-year follow-up. In addition, absolute values of the KOOS Pain and Symptoms subscales were analyzed at 2-year follow-up.

### Predictor Variables

The predictor variables of interest were analyzed as recorded by the New Zealand ACL Registry through the preoperative patient questionnaire and the operative data form completed by the surgeon. This included patient age, sex, time from injury to surgery, history of knee surgery, graft type (BTB vs hamstring tendon autograft), concomitant anterolateral ligament reconstruction or lateral extra-articular tenodesis, any injury or treatment to the medial or lateral meniscus (no injury/no treatment, resection, or repair), a grade 3 or 4 cartilage injury documented at the time of primary ACL reconstruction according to the International Cartilage Regeneration & Joint Preservation Society, and significant knee pain or kneeling difficulty before surgery. The Marx score of patients was also analyzed to compare the activity levels between patients reporting CKP or SKD versus those without such complications.

### Statistical Analysis

Descriptive statistics were provided as mean values with standard deviations or median values with interquartile ranges. Continuous variables were assessed for normality through the visualization of Q-Q plots and histograms. Univariate analysis was performed using the chi-square test. Multivariate analysis was performed via binary logistic regression to compute odds ratios (ORs) with 95% CIs. All variables including age, sex, time to surgery, previous surgery, concurrent anterolateral ligament reconstruction or lateral extra-articular tenodesis, meniscal treatment, chondral injury, and preoperative CKP or SKD were entered into a forward stepwise regression model to identify independent predictors of postoperative CKP and SKD. Absolute values of the KOOS Pain and Symptoms subscales were compared via the Mann-Whitney *U* test. Results were considered statistically significant at *P* < .05. All analyses were performed using SPSS Statistics (Version 25; IBM).

## Results

A total of 10,999 patients had a minimum follow-up of 6 months and were eligible for analysis (Table 1). The mean follow-up duration of this cohort was 3.52 ± 1.85 years. The patient response rate for both primary outcomes was 63%, 55%, and 41% at 6-month, 1-year, and 2-year follow-up, respectively. Overall, 56.6% of patients were male, with a mean age of 30 years. The median time from injury to surgery was 4.4 months, with 64.6% undergoing surgery within 6 months. The BTB autograft was used in 26.6% of patients. In terms of meniscal treatment, 21% of patients underwent either medial or lateral resection, while 19.0% underwent repair of a medial meniscal tear, and 15.0% underwent repair of the lateral meniscus. A grade 3 to 4 chondral lesion in any compartment was reported in 9.7% of patients. Finally, 45.1% of patients reported CKP and 34.4% reported SKD before their primary ACL reconstruction.

**Table 1:** Univariate Analysis of Consequential Knee Pain^*a*^

		6 Months			1 Year			2 Years		
	Total, n	No. of Responses	Yes, n (%)	*P* Value	No. of Responses	Yes, n (%)	*P* Value	No. of Responses	Yes, n (%)	*P* Value
Primary ACL reconstruction	10,999	6897	1097 (15.9)		6038	702 (11.6)		4492	420 (9.3)	
Sex				<.001			.568			.446
Male	6224	3500	503 (14.4)		3063	349 (11.4)		2240	202 (9.0)	
Female	4775	3397	594 (17.5)		2975	353 (11.9)		2252	218 (9.7)	
Age, y				<.001			<.001			.008
≤20	2689	1573	158 (10.0)		1313	94 (7.2)		932	69 (7.4)	
21-30	4162	2477	370 (14.9)		2174	227 (10.4)		1581	138 (8.7)	
≥31	4148	2847	569 (20.0)		2551	381 (14.9)		1979	213 (10.8)	
Time to surgery, mo				.656			.376			.314
<6	7108	4474	704 (15.7)		3921	439 (11.2)		2902	265 (9.1)	
6-12	2259	1403	220 (15.7)		1233	155 (12.6)		908	81 (8.9)	
>12	1616	1008	170 (16.9)		877	106 (12.1)		678	74 (10.9)	
NR	16	12	3 (25.0)		7	2 (28.6)		4	0 (0.0)	
Previous surgery				.069			.004			<.001
Yes	495	317	62 (19.6)		304	51 (16.8)		259	42 (16.2)	
No	10,504	6580	1035 (15.7)		5734	651 (11.4)		4233	378 (8.9)	
Graft choice				.164			.230			.494
BTB	2927	1770	300 (16.9)		1434	154 (10.7)		968	96 (9.9)	
Hamstring tendon	8072	5127	797 (15.5)		4604	548 (11.9)		3524	324 (9.2)	
ALL reconstruction/LET				.311			.230			.966
Yes	330	206	38 (18.4)		162	14 (8.6)		76	7 (9.2)	
No	10,669	6691	1059 (15.8)		5876	688 (11.7)		4416	413 (9.4)	
Medial meniscus				.029			.455			.012
No injury/no treatment	6604	4257	641 (15.1)		3707	420 (11.3)		2823	236 (8.4)	
Resection	2308	1388	230 (16.6)		1254	145 (11.6)		954	105 (11.0)	
Repair	2087	1252	226 (18.1)		1077	137 (12.7)		715	79 (11.0)	
Lateral meniscus				.624			.234			.797
No injury/no treatment	7060	4530	713 (15.7)		4001	457 (11.4)		2999	277 (9.2)	
Resection	2287	1361	228 (16.8)		1222	158 (12.9)		939	93 (9.9)	
Repair	1652	1006	156 (15.5)		815	87 (10.7)		554	50 (9.0)	
Grade 3-4 cartilage injury				<.001			<.001			.002
Yes	1062	664	146 (22.0)		579	93 (16.1)		445	59 (13.3)	
No	9672	6071	919 (15.1)		5310	584 (11.0)		3920	345 (8.8)	
NR	265	162	32 (19.8)		149	25 (16.8)		127	16 (12.6)	
Preoperative KOOS Pain ≤72				<.001			<.001			<.001
Yes	4962	2943	787 (26.7)		2543	503 (19.8)		1887	304 (16.1)	
No	5651	3770	271 (7.2)		3337	176 (5.3)		2493	104 (4.2)	
NR	386	184	39 (21.2)		158	23 (14.6)		112	12 (10.7)	

a ACL, anterior cruciate ligament; ALL, anterolateral ligament; BTB, bone–patellar tendon–bone; KOOS, Knee injury and Osteoarthritis Outcome Score; LET, lateral extra-articular tenodesis; NR, not recorded.

### CKP and SKD After Primary ACL Reconstruction

The overall incidence of CKP was highest at 6-month follow-up, with 15.9% of patients reporting a KOOS Pain subscore of ≤72 points (Table 1). The incidence decreased to 11.6% and 9.3% at 1- and 2-year follow-up, respectively. Similarly, the incidence of SKD was highest at 6-month follow-up, affecting 21.3% of all patients (Table 2). At 1- and 2-year follow-up, the incidence decreased to 14.3% and 12.0%, respectively.

**Table 2:** Univariate Analysis of Severe Kneeling Difficulty^*a*^

		6 Months			1 Year			2 Years		
	Total	No. of Responses	Yes, n (%)	*P* Value	No. of Responses	Yes, n (%)	*P* Value	No. of Responses	Yes, n (%)	*P* Value
Primary ACL reconstruction	10,999	6846	1461 (21.3)		6010	862 (14.3)		4471	537 (12.0)	
Sex				<.001			<.001			.038
Male	6224	3468	627 (18.1)		3048	382 (12.5)		2228	245 (11.0)	
Female	4775	3378	834 (24.7)		2962	480 (16.2)		2243	292 (13.0)	
Age, y				<.001			.484			.629
≤20	2689	1563	287 (18.4)		1310	177 (13.5)		926	111 (12.0)	
21-30	4162	2459	569 (23.1)		2165	307 (14.2)		1579	199 (12.6)	
≥31	4148	2824	605 (21.4)		2535	378 (14.9)		1966	227 (11.5)	
Time to surgery, mo				.032			.576			.684
≤6	7108	4439	989 (22.3)		3898	559 (14.3)		2887	354 (12.3)	
6-12	2259	1393	279 (20.0)		1233	168 (13.6)		903	101 (11.2)	
>12	1616	1002	191 (19.1)		872	133 (15.3)		677	82 (12.1)	
NR	16	12	2 (16.7)		7	2 (28.6)		4	00 (0.0)	
Previous surgery				.521			.109			.002
Yes	495	316	72 (22.8)		303	53 (17.5)		260	47 (18.1)	
No	10,504	6530	1389 (21.3)		5707	809 (14.2)		4211	490 (11.6)	
Graft choice				<.001			<.001			<.001
BTB	2927	1757	572 (32.6)		1428	333 (23.3)		968	206 (21.3)	
Hamstring tendon	8072	5089	889 (17.5)		4582	529 (11.5)		3503	331 (9.4)	
ALL reconstruction/LET				.005			.914			.688
Yes	330	205	60 (29.3)		164	24 (14.6)		76	8 (10.5)	
No	10,669	6641	1401 (21.1)		5846	838 (14.3)		4395	529 (12.0)	
Medial meniscus				.003			.008			.408
No injury/no treatment	6604	4229	884 (20.9)		3694	518 (14.0)		2812	326 (11.6)	
Resection	2308	1378	270 (19.6)		1245	160 (12.9)		950	116 (12.2)	
Repair	2087	1239	307 (24.8)		1071	184 (17.2)		709	95 (13.4)	
Lateral meniscus				.036			.693			.128
No injury/no treatment	7060	4502	933 (20.7)		3979	571 (14.4)		2980	340 (11.4)	
Resection	2287	1344	284 (21.1)		1217	181 (14.9)		940	118 (12.6)	
Repair	1652	1000	244 (24.4)		814	110 (13.5)		551	79 (14.3)	
Grade 3-4 cartilage injury				.032			.415			.425
Yes	1062	659	162 (24.6)		573	88 (15.4)		446	58 (13.0)	
No	9672	6030	1265 (21.0)		5289	746 (14.1)		3901	457 (11.7)	
NR	265	157	34 (21.7)		148	28 (18.9)		124	22 (17.7)	
Preoperative kneeling difficulty				<.001			<.001			<.001
Yes	3788	2267	765 (33.7)		1990	460 (23.1)		1491	292 (19.6)	
No	6031	3922	548 (14.0)		3440	315 (9.2)		2577	203 (7.9)	
NR	1180	657	148 (22.5)		580	87 (15.0)		403	42 (10.4)	

a ACL, anterior cruciate ligament; ALL, anterolateral ligament; BTB, bone–patellar tendon–bone; LET, lateral extra-articular tenodesis; NR, not recorded.

### BTB Versus Hamstring Tendon Autograft

**Figure 1.**
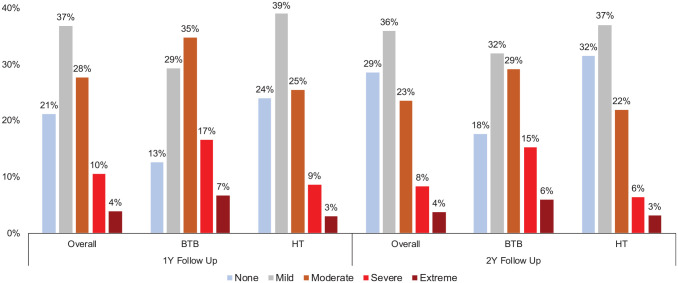
Distribution of responses to difficulty with kneeling. BTB, bone–patellar tendon–bone; HT, hamstring tendon.

At 2-year follow-up, 21.3% of patients with a BTB autograft reported SKD compared with 9.4% of patients with a hamstring tendon autograft (*P* < .001) (Table 2). On multivariate analysis, the use of a BTB autograft was the most important risk factor for SKD at all 3 follow-up time points (adjusted OR, 2.88, 3.06, and 3.12, respectively; *P* < .001). However, there was no difference between the BTB and hamstring tendon autografts in the rate of CKP (9.9% vs 9.2%, respectively; *P* = .494) or in absolute values of the KOOS Pain (mean, 88.7 ± 13.1 vs 89.0 ± 13.1, respectively; *P* = .37) and KOOS Symptoms (mean, 82.5 ± 15.0 vs 82.1 ± 15.4, respectively; *P* = .49) subscales at 2-year follow-up. The distribution of responses to the extent of difficulty with kneeling reported by patients at 1- and 2-year follow-up is displayed in Figure 1, demonstrating that a higher proportion of patients with a BTB autograft reported “severe” or “extreme” difficulty with kneeling compared with patients with a hamstring tendon autograft.

### Other Factors Associated With CKP

The most important risk factor for CKP after ACL reconstruction at all 3 follow-up time points was the presence of CKP before surgery (adjusted OR, 4.38, 4.06, and 4.10, respectively; *P* < .001) (Table 3). Age was the second most important risk factor, with patients aged ≥31 years having higher odds of knee pain at 6-month (adjusted OR, 1.73; *P* < .001) and 1-year (adjusted OR, 1.56; *P* = .001) follow-up. Female sex and a grade 3 to 4 chondral lesion were predictive of knee pain at 6 months postoperatively but were not associated with knee pain at 1- or 2-year follow-up.

**Table 3:** Multivariate Analysis: Independent Predictors for Consequential Knee Pain^*a*^

	6 Months		1 Year		2 Years	
	OR (95% CI)	*P* Value	OR (95% CI)	*P* Value	OR (95% CI)	*P* Value
Sex						
Male	Reference					
Female	1.17 (1.01-1.35)	.035				
Age, y						
≤20	Reference		Reference			
21-30	1.38 (1.11-1.71)	.004	1.30 (1.00-1.70)	.055		
≥31	1.73 (1.39-2.15)	<.001	1.56 (1.20-2.04)	.001		
Graft choice						
BTB	1.37 (1.16-1.62)	.042				
Hamstring tendon	Reference					
Preoperative KOOS Pain ≤72						
Yes	4.38 (3.75-5.11)	<.001	4.06 (3.37-4.90)	<.001	4.10 (3.23-5.19)	<.001
No	Reference		Reference		Reference	
Marx score	0.93 (0.91-0.95)	<.001	0.93 (0.91-0.95)	<.001	0.92 (0.90-0.94)	<.001
Medial meniscus						
No injury/no treatment	Reference		Reference			
Resection	0.82 (0.68-0.99)	.042	0.78 (0.62-0.97)	.023		
Repair	1.23 (1.02-1.48)	.027	1.17 (0.94-1.46)	.173		
Grade 3-4 cartilage injury						
Yes	1.35 (1.09-1.68)	.006				
No	Reference					

a BTB, bone–patellar tendon–bone; KOOS, Knee injury and Osteoarthritis Outcome Score; OR, odds ratio.

### Other Factors Associated With SKD

Patients who reported SKD before surgery had higher odds of SKD after surgery at all 3 follow-up time points (adjusted OR, 3.28, 2.96, and 2.90, respectively; *P* < .001) (Table 4). In addition, female patients had higher odds of SKD at 6-month (adjusted OR, 1.47; *P* < .001) and 1-year (adjusted OR, 1.23; *P* < .016) follow-up. At 6-month follow-up, higher odds of SKD were observed in patients aged 21 to 30 years (adjusted OR, 1.33; *P* = .002) and in patients with a concomitant grade 3 to 4 chondral lesion (adjusted OR, 1.28; *P* < .001), but lower odds of SKD were observed in patients who had undergone medial meniscal resection (adjusted OR, 0.79; *P* = .009).

**Table 4:** Multivariate Analysis: Independent Predictors for Severe Kneeling Difficulty^*a*^

	6 Months		1 Year		2 Years	
	OR (95% CI)	*P* Value	OR (95% CI)	*P* Value	OR (95% CI)	*P* Value
Sex						
Male	Reference		Reference			
Female	1.47 (1.28-1.67)	<.001	1.23 (1.04-1.44)	.016		
Age, y						
≤20	Reference					
21-30	1.33 (1.11-1.59)	.002				
≥31	1.16 (0.96-1.41)	.127				
Graft choice						
BTB	2.88 (2.49-3.33)	<.001	3.06 (2.57-3.65)	<.001	3.12 (2.52-3.87)	<.001
Hamstring tendon	Reference		Reference		Reference	
Preoperative kneeling difficulty						
Yes	3.28 (2.87-3.75)	<.001	2.96 (2.51-3.49)	<.001	2.90 (2.37-3.54)	<.001
No	Reference		Reference		Reference	
Marx score	0.92 (0.90-0.94)	<.001	0.92 (0.91-0.94)	<.001	0.93 (0.92-0.95)	<.001
Medial meniscus						
No injury/no treatment	Reference					
Resection	0.79 (0.66-0.94)	.009				
Repair	1.09 (0.92-1.30)	.316				
Grade 3-4 cartilage injury						
Yes	1.28 (1.03-1.59)	<.001				
No	Reference					

a BTB, bone–patellar tendon–bone; OR, odds ratio.

### Association Between Activity Levels and CKP and SKD

On both univariate and multivariate analyses, patients who reported CKP or SKD also reported lower activity levels at all 3 follow-up time points (Tables 3 and 4). Patients without CKD or SKD reported a mean Marx score of 7.1 and 7.0, respectively, at 2-year follow-up. Although patients with CKD or SKD reported lower mean Marx scores of 5.0 and 5.6, respectively, these scores indicate that they were still active and participating in sports.

## Discussion

The most important finding of this study was that the BTB autograft was associated with difficulty with kneeling after ACL reconstruction, but it did not result in a more painful or symptomatic knee compared with the hamstring tendon autograft.

This study analyzed 2 different outcomes to differentiate significant knee pain from significant difficulty with kneeling. A study from the MOON group proposed a definition of significant knee pain as a KOOS Pain subscore of ≤72 points, which it found in 9% of patients at 2- and 6-year follow-up.^34^ The present study used the same cutoff to define CKP and found an incidence of 15.9% at 6-month follow-up and 9.3% at 2-year follow-up. There are a number of potential causes for knee pain after ACL reconstruction, including a repeat injury, donor-site morbidity, pain at the skin incision site, pain related to hardware fixation, osteoarthritis, and quadriceps muscle weakness.^12,30,34^ Although this study was unable to identify the causes for postoperative knee pain, patients who reported knee pain before undergoing surgery had 4 times the odds of reporting knee pain after surgery. Interestingly, Ware et al^33^ performed a prospective study of 72 patients undergoing ACL reconstruction and found that lower preoperative KOOS scores were strongly associated with a more painful and symptomatic knee at 7-year follow-up.

Graft choice is the most widely debated topic in ACL reconstruction, and it is unclear whether it is a risk factor for knee pain. Anterior knee pain has been associated with the BTB autograft^1,3,6,12,26,28,35^; however, there is variation in how studies define anterior knee pain and subsequently a lack of clarification on whether this is disabling pain that impairs function or whether it could be an alternative description for difficulty with kneeling.^15^ Our study found that patients with a BTB autograft were 3 times more likely to report SKD but were not more likely to suffer from significant knee pain or symptoms compared with patients with a hamstring tendon autograft. Other studies have also attempted to differentiate knee pain from kneeling difficulty. In a randomized study of 72 patients performed by Aune et al,^2^ no statistically significant difference in knee pain was found between graft types at 2-year follow-up (16.1% vs 12.5%, respectively; *P* > .05); however, when patients were asked on a visual analog scale to rate their discomfort when kneeling, 35.5% of patients with BTB grafts reported kneeling problems compared with 18.9% of patients with hamstring tendon grafts (*P* < .05). In a prospective cohort study of 958 cases, Rousseau et al^26^ found that anterior knee pain was more common in patients with BTB grafts in the first 2 years after surgery (23.3% vs 12.6%, respectively; *P* < .001), but there was no difference after 2 years of follow-up (3.1% vs 2.5%, respectively; *P* = .63). Last, the Swedish National ACL Register also analyzed absolute values of the KOOS between BTB and hamstring tendon autografts.^29^ Both the Swedish and the New Zealand registries have found equivalent scores between graft types for all 5 KOOS subscales at 2-year follow-up, including the Pain and Symptoms subscales.^21,29^ Surgeons in various centers have preferred the hamstring tendon autograft because of the association between the BTB autograft and donor-site morbidity. However, it is possible that donor-site morbidity associated with the BTB autograft may not be as clinically significant as previously thought, given the variation and inconsistencies in how it is defined in the literature. Furthermore, these data suggest that there is no difference in overall pain or symptoms between graft types, and therefore, this should not prevent clinicians from using a BTB autograft.

An interesting finding from this study was the lower activity levels reported by patients who had CKP or SKD. Although having a painful and symptomatic knee may discourage patients or make them less able to participate in sports, it is unclear why patients who find it difficult to kneel reported lower activity levels. In this study, patients with a BTB autograft were 3 times more likely to report SKD; however, previous studies analyzing the same patients from the New Zealand ACL Registry found that patients with a BTB autograft had higher Marx scores (mean, 7.7 vs 6.3, respectively; *P* < .001)^22^ and were more likely to return to their preinjury activity level (adjusted OR, 1.63; *P* = .008) compared with patients with a hamstring tendon autograft.^21^ Regardless, the findings of this study support the importance of individualizing graft choice before ACL reconstruction. All patients considering ACL reconstruction should be informed of the potential complications as well as the advantages and disadvantages of each graft type. The higher rate of SKD after surgery when using a BTB autograft is a complication that should be considered in select patient populations such as those who may be required to kneel as part of their occupation or sport. In these certain patient populations, the hamstring tendon autograft may be the more appropriate graft choice because of the lower rate of kneeling difficulty. However, the BTB autograft remains the ideal graft choice for athletes undergoing ACL reconstruction, as it provides the highest chance of returning to sport^21^ with the lowest risk of reruptures.^7,11,14,22^

### Limitations

The main limitation of this study is the use of only 1 type of patient-reported outcome measure: the KOOS questionnaire. As the KOOS was initially designed for use in patients with knee osteoarthritis, several studies have validated its usefulness in ACL reconstruction.^4,16^ Currently, there is no gold standard patient-reported outcome measure for ACL reconstruction.^9,13^ However, in this study, the KOOS was not used to assess the success of ACL reconstruction but rather as a tool to compare outcomes between graft types. Second, the patient response rate to the KOOS in this study was 63%, 55%, and 41% at 6-month, 1-year, and 2-year follow-up, respectively. Although loss to follow-up may introduce selection bias, a previous study from the Danish Knee Ligament Reconstruction Registry found no difference in KOOS scores between responders and nonresponders.^23^ Furthermore, a similar study from the Swedish National ACL Register analyzed KOOS scores after ACL reconstruction and comparably had a patient response rate of 30% at both 1- and 2-year follow-up.^29^ Last, although a strength of registry studies is the availability of prospective data on a large patient cohort, they are limited in their ability to only report associations. They are not able to thoroughly investigate the reason for each association and cannot infer causality. In this study, the potential effect of hardware and implants in contributing to pain was not able to be investigated. It was also not able to identify the reasons for the higher rate of SKD in patients with a BTB autograft. Future randomized controlled trials are needed to further compare the incidence of knee pain versus difficulty with kneeling when comparing graft types and investigate the contributing factors for both outcomes.

## Conclusion

At 2-year follow-up after primary ACL reconstruction, 9.3% of patients reported CKP, and 12.0% reported SKD. The BTB autograft was associated with difficulty with kneeling, but it did not result in a more painful or symptomatic knee compared with the hamstring tendon autograft.

## Footnotes

¶ References 5, 10, 15, 19, 20, 25, 27, 28, 32.
